# Multivariate modelling of milk fatty acid profile to discriminate the forages in dairy cows’ ration

**DOI:** 10.1038/s41598-021-02600-9

**Published:** 2021-12-01

**Authors:** Giorgia Riuzzi, Hannah Davis, Ilaria Lanza, Gillian Butler, Barbara Contiero, Flaviana Gottardo, Severino Segato

**Affiliations:** 1 Department of Animal Medicine, Production and Health, University of Padova, Viale dell’Università, 16, 35020 Legnaro, PD Italy; 2 School of Natural and Environmental Science, Newcastle University, Newcastle Upon Tyne, NE1 7RU UK

**Keywords:** Biochemistry, Chemical biology, Plant sciences

## Abstract

Although there are many studies on the importance of fatty acids (FA) in our diet and on the influence of dairy diets on FA metabolism, only a few investigate their predictive capacity to discriminate the type, amount and conservation method of farm forages. This research quantifies differences in milk FA concentrations and, using a supervised factorial discriminant analysis, assesses potential biomarkers when replacing maize with other silages, grass/lucerne hays or fresh grass. The statistical modelling identified three main clusters of milk FA profiles associated with silages, hays and fresh grass as dominant roughages. The main implication of a dairy cow feeding system based on poliphytic forages from permanent meadows is enhancing milk’s nutritional quality due to an increase in beneficial omega-3 polyunsaturated FA, conjugated linoleic acids and odd chain FA, compared to feeding maize silage. The study also identified a small but powerful and reliable pool of milk FA that can act as biomarkers to authenticate feeding systems: C16:1 *c*-9, C17:0, C18:0, C18:3 *c*-9, *c*-12, *c*-15, C18:1 *c*-9, C18:1 *t*-11 and C20:0.

## Introduction

Dairy products contain a number of different types of lipid which are dominated by triacylglycerols, potentially comprising of over 400 different individual fatty acids (FA), of varying length and saturation. There is a small fraction of other lipids like vitamins, phospholipids and glycerolipids^1^. Numerous studies consider milk FA and vitamin content especially those deemed relevant for health or contributing to organoleptic properties^1–3^. Despite a relatively high proportion of FA thought to have a negative impact on health (creating some concernes on dairy consumption), research actually suggests milk consumption has a positive health effect, thanks to short chain (≤ C10) FA (SCFA), conjugated linoleic acids (CLA), omega-3 (n-3) polyunsaturated FA (PUFA) and odd- and branched-chain FA (OCFA and BCFA)^1,4,5^. SCFA have been shown to have antiviral activities and delay tumors’ growth^6,7^, CLA and n-3 have numerous beneficial functions for human health^2^ and there is increasing interest in milk OCFA and BCFA (mainly derived from rumen bacteria) reported to have anticarcinogenic effects^8^.

The FA profile of milk is extremely variable, depending on genetics, season and stage of lactation^9^, although feed management is recognised as having the strongest influence^10,11^. Many studies report the impact of dairy feeding on milk FA composition from intensive lowland production^9,12,13^. Under these systems, cows fed forage from diverse meadows produce milk which is richer in beneficial FA such as CLA and n-3 compared with maize silage diets. This influence is stronger for grazing animals^13^ but also seen for mixed diets with forage from such pastures^9^. The role of forage type and conservation method, especially ensiling, on rumen FA metabolism is under debate^12,14–16^, however, most studies have been carried out under controlled experimental conditions. It is unclear if they represent the challenges and variation seen on commercial farms where potentially contrasting effects of genetics, rearing, feeding and other management, work on metabolic pathways in synergy^11,17^. O’Callaghan et al*.*^13^ report relatively high concentrations of C18:2n-6, C18:3n-6, C22:0, C22:1n-9 and C18:2 *c*-10, *t*-12 in milk from both grass hay and maize silage diets, when supplemented with high levels of concentrate feeds. However, increasing the proportion of fresh or ensiled poliphytic (mixed or diverse) forages in dairy diets leads to significantly more n-3 and CLA as well as vaccenic acid (VA, C18:1 *t*-11), even if cows are housed and fed a total mixed ration (TMR)^9,12^. This extensive knowledge on the influence of forages on bovine FA metobolism, has generated an interest and perspective on the potential application of multifactorial models to link milk FA and the botanical origin, conservation method and dietary proportion of forages^9,18^. Supervised multivariate models might predict the impact of dietary forage on metabolic pathways, from the rumen to the mammary gland, responsible for FA release into milk^19,20^. Furthermore, a chemometric approach, based on a pattern recognition supervised modelling, can determine a functional relationship between the analytes (i.e., FA) and the predictors (i.e., forage type), identifying useful features which discriminate between classes^21,22^.

This research quantifies differences in milk FA profiles from replacing maize silage with (a) silages from other cereal or legume crops, (b) grass and lucerne hays or (c) fresh grass, using supervised factorial discriminant analysis to verify if differences in FA can be used to fingerprint dairy production chains. Moreover, a linear regression model and clustering of the variability by a set of descriptive statistics were performed to predict milk FA profile in relation to dietary forage.

## Materials and methods

### Ethical statement and experimental design

The study did not influence farm activities or management strategies and did not involve invasive procedures or manipulation of lacating dairy cows. Since the impact on the animals’ welfare was negligible, ethical review and approval from the local or national ethics committee was unnecessary. With farmer consent, a qualified veterinarian collected records, feed (pre and post feeding) and bulk tank milk samples from 14 commercial dairy farms in the middle of the Italian lowland area, Po Valley (North East of Italy, 45° 19′ 49″ N 9° 47′ 56″ E). The farms were selected to represent average herd size and milk yield characterizing the Italian intensive dairy system^23,24^ and all were affiliated to Regional Breeders’ Association, ensuring descriptive characteristics were recorded monthly over the experimental period (Table 1). Records were collected on diet details and milk production at each sampling visit (5 *per* farm) and averaged *per* lactating cow *per* day. On this basis, the average dry matter intake (DMI) was calculated by difference between amount of total mixed rations (TMR) distributed to the lactating cows and refusals after 24 h or before the subsequent distribution. Milk production was standardised to ‘Fat Protein Corrected Milk’ (FPCM) as per International Dairy Federation (IDF)^25^ using the following equation:

$${\text{FPCM }}\left( {{\text{kg}}\,{{per}}\,{\text{day}}} \right) = {\text{Y}} \times [(0.0{929} \times {\text{F}} + 0.0{588} \times {\text{TP}} + 0.{192})/0.{7576}]$$

where Y, milk yield as kg*/*day; F, fat as percentage; TP, true protein as percentage (= CP × 0.93, and CP as N_Kjeldahl_ × 6.38); 0.0929, 0.0588 and 0.7576 are Mcal*/*kg of F, TP and standardized milk (4.0% F and 3.3% TP), respectively.

**Table 1:** Herd descriptive statistics (average ± SD); diet formulation (%) and proximate composition (% on DM) of the five feeding groups according to the main roughage source.

	Dietary forage group				
	High Maize Silage	Medium Maize Silage	Mixed Crop Silages	Grass and lucerne hays	Green Grass
	HMS (*n* = 20)	MMS (*n* = 18)	MCS (*n* = 11)	HAY (*n* = 12)	GRG (*n* = 9)
**Herd descriptive statistics**					
Lactating cows (n)	96 (± 44)	122 (± 41)	68 (± 16)	71 (± 16)	50 (± 7)
Days in milk (d)	198 (± 29)	177 (± 27)	172 (± 17)	165 (± 21)	189 (± 24)
Calving interval (d)	434 (± 31)	408 (± 17)	410 (± 24)	399 (± 17)	403 (± 17)
**Diet ingredients (% DM)**					
Maize silage	35 (± 5)	23 (± 4)	0	0	0
Other silages	6 (± 4)	15 (± 5)	41 (± 6)	6 (± 4)	11 (± 9)
Permanent meadow hay	8 (± 4)	8 (± 6)	8 (± 4)	35 (± 10)	19 (± 12)
Lucerne hay	3 (± 2)	4 (± 2)	2 (± 2)	13 (± 4)	6 (± 4)
Fresh grass	0	0	0	0	25 (± 5)
Energetic concentrates	27 (± 4)	27 (± 7)	34 (± 6)	35 (± 8)	27 (± 7)
Protein concentrates	16 (± 5)	19 (± 6)	11 (± 4)	8 (± 5)	9 (± 5)
Residual	5 (± 2)	4 (± 2)	4 (± 2)	3 (± 2)	3 (± 1)
**Diet composition (% DM)**					
DM (%)	54.8 (± 5.3)	56.6 (± 5.1)	55.8 (± 6.5)	69.5 (± 5.8)	64.7 (± 8.6)
Crude protein	14.0 (± 0.5)	14.1 (± 0.6)	14.1 (± 0.6)	14.0 (± 1.1)	13.5 (± 1.3)
Ether extract	2.7 (± 0.4)	2.8 (± 0.4)	2.7 (± 0.5)	2.6 (± 0.4)	2.5 (± 0.7)
Ash	8.1 (± 0.7)	7.6 (± 0.5)	8.3 (± 0.4)	7.9 (± 0.6)	7.8 (± 0.4)
aNDF	36.8 (± 1.9)	37.2 (± 2.1)	37.7 (± 3.1)	40.7 (± 4.4)	37.6 (± 4.0)
ADF	21.7 (± 1.6)	22.2 (± 1.3)	22.3 (± 1.9)	23.9 (± 1.6)	20.3 (± 2.9)
Starch	22.6 (± 1.6)	22.4 (± 2.3)	21.7 (± 3.1)	19.4 (± 1.1)	20.6 (± 4.7)

*Other silages*, sorghum, wheat, lucerne, grass, ryegrass; *energetic concentrates*, maize products, sorghum mash, barley meal; *protein concentrates*, soybean products, sunflower meal; *residual*, straw, bran, beet pulps, salts, mineral-vitamin premix; *aNDF*, neutral detergent fibre; *ADF*, acid detergent fibre.

The experimental protocol allocated each farm record to one of five feeding groups (FG), based on the main roughage source (% of TMR on dry matter basis): (i) high maize silage (HMS; maize silage ≥ 32%); (ii) medium maize silage (MMS; maize silage = 12–26%); (iii) mixed crop silages (MCS; other crop silages ≥ 37% and maize silage = 0%); (iv) grass and lucerne hays (HAY; permanent meadow and lucerne hays ≥ 42%, maize silage = 0%, other crop silages < 9%); (v) green grass (GRG; fresh grass > 20% and maize silage = 0%). All forages were home-produced. Maize silage (late variety, such as FAO class 600–700) and the permanent meadows (mix of perennial ryegrass, meadow fescue with a minor presence of red and white clover) were produced in optimal pedoclimatic and irrigated conditions with an average annual yield of 21 and 10 t DM/ha, respectively. The third main fodder, used in the MCS group, was a mix of ensiled forages such as sorghum (25%), lucerne (25%), wheat (20%), perennial grass (15%) and Italian ryegrass (15%), with a medium–high productive yield. All herds (including the GRG group) were fed TMR, formulated to cover the herd’s energy and protein requirements, based on NRC standard^26^. Average rations for the five FG (% on DM) and their proximate compositions (% on DM) are reported in Table 1.

### Sample collection and chemical analysis

In 2018, five raw bulk milk samples were collected from each farm in March, May, July, September and December (*n* = 70) and at each visit, the current TMR was also sampled and formulations recorded. Since it is not uncommon for farms to alter diet ingredients due to seasonal feed supply, some farms changed TMR formulation over the experimental period, essentially changing group. In details: HMS covered 4 farms and *n* records = 20; MMS, 4 farms and *n* = 18 (because one original MMS farm changed twice, once into MCS and once into HAY); MCS, 2 farms and *n* = 11 (because one switched from MMS); HAY, 2 farms and *n* = 12 (because one switched from MCS and another one from HAY); GRG, 2 farms and *n* = 9 (because one orginal GRG farm changed once into a HAY diet). However, according to Rego et al.^14^, we ensured at least three weeks between diet change and milk sampling. TMR and milk sub-samples were frozen at − 20 °C until analysis. After thawing, TMR samples were analysed for chemical traits using the AOAC procedures (#934.01 for dry matter DM^27^; #2001.11 for crude protein, CP^28^; #2003.05 for ether extract, EE^29^; #942.05 for ash^27^ and #996.11 for starch^30^) and ANKOM technology for neutral detergent fiber (aNDF)^31^ and acid detergent fiber (ADF)^32^.

The milk proximate composition (crude protein, casein, fat, lactose) and chemical traits (urea, pH) were recorded by a Fourier transform mid-infrared (FT-MIR) spectroscopy technique using a MilkoScan FT6000 (Foss Electric A/S, Hillerød, Denmark). Additionally, the somatic cell count (SCC, 100,000/ml) was performed by a Fossomatic 5000 (Foss Electric A/S, Hillerød, Denmark) and reported as SCC score calculated with the following formula [log2 (SCC/100,000) + 3].

For milk FA analysis, 2 replicates of approximately 35 g from each sample were freeze-dried, mixed to a fine homogenous powder and transferred to suitable vials. These lyophilized samples were methylated and esterified to prepare for gas chromatography (GC), as described by Chilliard et al.^33^ and Stergiadis et al.^34^. The chemicals used for extraction of FA, correction for SCFA, analytical standards and identification of peaks followed the methodology of Stergiadis et al.^35^. To optimize peak separation, modifications to the chromatographic conditions from the original method by Chilliard et al*.*^33^ was followed, as reported by Stergiadis et al*.*^35^. FA results are expressed as g/100 g of total quantified FA. Values for individual FA were used to calculate total saturated FA (SFA), short chain (≤ C10) FA (SCFA), monounsaturated FA (MUFA), polyunsaturated FA (PUFA), conjugated linoleic acids (CLA), highly unsaturated (≥ 4 double bonds) FA (HUFA), odd chain FA (OCFA), n-3 (omega-3 FA), n-6 (omega-6 FA), HUFAn-3 as well as n-3:n-6 and n-6:n-3 ratio.

### Statistical analysis

All analyses were carried out using the SAS 9.4 software (SAS Institute Inc., Cary, NC, USA) and XLStat (Addinsoft, release 2016, New York, USA). Herd performances (DMI and milk production expressed *per* cow *per* day) and raw bulk milk chemical and FA profile data were analysed using a linear mixed model that included the fixed effects of feeding group (FG: i-v) and the random effect of the farm (SAS PROC MIXED). Pairwise comparisons among levels of the FG factor were performed using Bonferroni correction. The hypotheses of the linear model on the residuals were graphically assessed.

The dataset of FA profiles was subjected to supervised multivariate factorial discriminant analysis (FDA), considering the FG as the predictor factor. The FDA split the total variance in four main canonical functions; F1-F4. The outcomes of the FDA were plotted to classify the five FG according to the first two main canonical functions F1 and F2. The correlation coefficients (with absolute value greater than 0.20) between the original FA and F1 and/or F2 were also plotted in the FDA-scattergram. The reliability of the FDA classification model was assessed by a leave one out cross-validation (SAS PROC DISCRIM). A confusion matrix was built throughout the results of the procedure and the classification performance was assessed using accuracy, precision, sensitivity, specificity and Matthews correlation coefficient (MCC)^36^.

A multiple stepwise regressions (SAS PROC REG) were preformed on the four main forages types (maize silage, mixed crop silages, grass and lucerne hays, fresh grass) on some FA and their derived chemical classes (SFA, MUFA, PUFA, CLA, HUFAn-3, OCFA). The regression coefficients were estimated. The most discriminative FA selected by the FDA were graphically represented by some box-whisker plots across the five FG.

## Results

### Dairy farm description, herd performance and milk quality

Mean herd characteristics of the five FG are reported in Table 1 showing major differences in herd size—farms using maize silage milked more cows than farms feeding dried or fresh grass/legume forage. Table 2 shows there were also significant differences between FG for both daily milk yield per cow and DMI (likely to be linked) as well as some aspects of proximate composition. The lowest daily FPCM yield was recorded for the GRG group, which also showed significantly lowest CP, casein and lactose concentrations.

**Table 2:** Effect of dietary roughage source (forage group) on DM intake (DMI), milk production, composition and quality traits.

	Dietary forage group					SEM	*p* value
	HMS (*n* = 20)	MMS (*n* = 18)	MCS (*n* = 11)	HAY (*n* = 12)	GRG (*n* = 9)		
DM intake (kg/day)	23.4^ab^	24.5^a^	23.4^ab^	22.6^ab^	21.5^b^	0.5	**0.001**
**Milk production (kg/day)**							
Milk yield	30.5^a^	31.5^a^	30.6^a^	29.1^ab^	26.2^b^	1.4	**0.003**
FPCM	30.3^a^	31.2^a^	30.2^a^	28.9^ab^	25.5^b^	1.3	**0.001**
**Milk composition (g/100 g)**							
Crude protein	3.52^a^	3.48^a^	3.46^ab^	3.47^a^	3.32^b^	0.05	**0.014**
Casein	2.71^a^	2.69^ab^	2.65^ab^	2.67^ab^	2.50^b^	0.05	**0.025**
Fat	4.21	3.91	3.99	3.87	3.84	0.12	0.083
Lactose	4.79^ab^	4.82^a^	4.78^ab^	4.74^ab^	4.71^b^	0.03	**0.034**
**Milk quality traits**							
SCC score (units)	3.98	3.74	3.75	3.98	4.37	0.20	0.140
Urea (mg/dL)	24.0	24.6	25.6	24.9	20.5	1.9	0.360
Native pH	6.65	6.67	6.65	6.65	6.65	0.01	0.147

*FPCM*, fat protein corrected milk (4.0% fat and 3.3% true protein); *SCC*, somatic cell count as log_2_ (SCC/100,000) + 3; *HMS*, high maize silage; *MMS*, medium maize silage; *MCS*, mixed crop silages; *HAY*, grass and lucerne hays; *GRG*, green grass; *SEM*, standard error of the means.

^a-b^LSMeans in a row without a common superscript differ (*p* < 0.05).

### Fatty acid profile

Table 3 reports mean concentration for each FG of abundant FA and those expected to be influenced by roughage sources although results for all 74 profiled FA are reported in a supplementary table. The FA profiles differed between FG for VA, C18:2 *c*-9, *t*-11 (CLA9), C20:5 *c*-5, *c*-8, *c*-11, *c*-14, *c*-17 (EPA), total CLA concentrations; all being higher (*p* < 0.05) for GRG than HMS milk and concentrations of SFA and SCFA were lower (*p* < 0.05). Differences also reached significance in comparing GRG and MMS milk for CLA9, total CLA and SCFA whereas for CLA9, GRG milk was significantly higher than for all other groups except HAY and SFA concentrations were lower than all groups except MMS. Differences were also significant in comparing GRG with MCS and HAY milk, where C16:0 (palmitic acid, PA) was lowest and PUFA concentrations highest in GRG milk. Other differences also existed when comparing HAY milk with the other groups; linoleic acid (LA, C18:2 *c*9, *c*12) had a tendancy (*p* = 0.066) to be lower than in GRG milk, and milk from the HAY group had more (*p* < 0.05) alpha linolenic acid (ALA, C18:3 *c*9, *c*12, *c*15) and total n-3 than HMS milk, resulting in a lower n-6:n-3 ratio (*p* < 0.05).

**Table 3:** Effect of dietary roughage source (forage group) on milk fatty acid (FA) profile (g/100 g of total quantified fatty acids).

Fatty acids	Dietary forage group					SEM	*p* value
	HMS (*n* = 20)	MMS (*n* = 18)	MCS (*n* = 11)	HAY (*n* = 12)	GRG (*n* = 9)		
C4:0	3.20^a^	2.97^ab^	3.01^ab^	3.10^ab^	2.73^b^	0.108	**0.024**
C6:0	2.30^a^	2.20^ab^	2.21^ab^	2.18^ab^	2.11^b^	0.046	**0.045**
C8:0	1.33	1.30	1.30	1.26	1.21	0.036	0.213
C10:0	2.99	3.03	2.92	2.76	2.70	0.102	0.122
C12:0	3.54	3.55	3.47	3.27	3.19	0.134	0.233
C14:0	11.6	11.8	11.7	11.6	11.2	0.260	0.468
C14:1 *c*-9	0.876	0.925	0.969	0.908	0.913	0.048	0.463
C15:0	1.09	1.17	1.22	1.13	1.09	0.065	0.384
C16:0 (PA)	32.7^ab^	32.2^ab^	33.3^a^	33.2^a^	31.2^b^	0.663	**0.026**
C16:1 *c*-9	1.67	1.65	1.71	1.71	1.84	0.095	0.505
C17:0	0.409	0.422	0.473	0.460	0.502	0.028	0.117
C18:0 (SA)	9.55	9.62	9.44	9.83	10.3	0.488	0.532
C18:1 *c*-9 (OA)	19.5	19.3	19.0	19.5	20.8	0.504	0.090
C18:1 *c*-12	0.268^a^	0.298^a^	0.243^ab^	0.167^b^	0.219^ab^	0.024	**0.002**
C18:1 *t*-10	0.302	0.317	0.262	0.274	0.356	0.051	0.239
C18:1 *t*-11 (VA)	0.702^b^	0.787^ab^	0.782^ab^	0.954^ab^	1.074^a^	0.085	**0.021**
ΣC18:1 *trans* (*t*-12+*t*-13 + *t*-14)	0.344^a^	0.315^ab^	0.266^ab^	0.241^b^	0.246^ab^	0.029	**0.037**
C18:2 *c*-9, *c*-12 (LA)	1.97^ab^	1.95^ab^	1.88^ab^	1.62^b^	2.13^a^	0.162	0.066
C18:2 *c*-9, *t*-11 (CLA9)	0.366^b^	0.409^b^	0.378^b^	0.442^ab^	0.619^a^	0.042	**0.001**
C18:2 *c*-15, *t*-11	0.067^b^	0.094^ab^	0.080^ab^	0.106^a^	0.110^a^	0.012	**0.041**
C18:3 *c*-9, *c*-12, *c*-15 (ALA)	0.343^b^	0.430^ab^	0.365^ab^	0.513^a^	0.487^ab^	0.048	**0.045**
C20:0	0.112	0.135	0.128	0.142	0.131	0.013	0.395
C20:4 *c*-5, *c*-8, *c*-11, *c*-14	0.141^ab^	0.165^a^	0.160^a^	0.116^b^	0.140^ab^	0.009	**0.007**
EPA	0.090^b^	0.097^ab^	0.091^ab^	0.124^a^	0.127^a^	0.010	**0.021**
C22:0	0.073	0.075	0.067	0.097	0.091	0.010	0.148
DHA	0.046	0.043	0.041	0.047	0.035	0.005	0.456
DPA	0.092	0.113	0.077	0.079	0.091	0.011	0.187
C23:0	0.034^b^	0.061^a^	0.051^ab^	0.050^ab^	0.051^ab^	0.007	**0.051**

Calculated values	Dietary forage group					SEM	*p* value
	HMS (*n* = 20)	MMS (*n* = 18)	MCS (*n* = 11)	HAY (*n* = 12)	GRG (*n* = 9)		
SFA	69.6^a^	69.1^ab^	69.9^a^	69.6^a^	66.9^b^	0.645	**0.003**
MUFA	25.7^ab^	25.8^ab^	25.5^b^	25.9^ab^	27.7^a^	0.547	**0.022**
PUFA	4.72^ab^	5.07^ab^	4.56^b^	4.55^b^	5.21^a^	0.216	**0.046**
HUFA	0.391	0.447	0.459	0.409	0.436	0.025	0.281
n-3	0.898^b^	1.08^ab^	0.979^ab^	1.219^a^	1.162^ab^	0.068	**0.003**
n-6	2.66	2.72	2.57	2.26	2.85	0.183	0.053
n-3:n-6	0.353^b^	0.436^ab^	0.401^ab^	0.570^a^	0.416^ab^	0.052	**0.018**
n-6:n-3	3.04^a^	2.53^ab^	2.67^ab^	2.11^b^	2.60^ab^	0.245	**0.044**
HUFAn-3	0.209	0.235	0.246	0.251	0.249	0.019	0.382
CLA	0.629^b^	0.687^ab^	0.558^b^	0.684^ab^	0.868^a^	0.048	**0.002**
SCFA	9.97^a^	9.60^a^	9.56^ab^	9.42^ab^	8.83^b^	0.205	**0.004**
OCFA	2.09	2.34	2.20	2.06	2.30	0.151	0.611

*HMS*, high maize silage; *MMS*, medium maize silage; *MCS*, mixed crop silages; *HAY*, grass and lucerne hays; *GRG*, green grass; *SEM*, standard error of the means.

Fatty acids abbreviations: *PA*, palmitic acid; *SA*, stearic acid; *OA*, oleic acid; *VA*, vaccenic acid; *LA*, linoleic acid; *CLA*, conjugated linoleic acid; *ALA*, alpha linolenic acid; *EPA*, eicosapentaenoic acid (C20:5 *c*-5, *c*-8, *c*-11, *c*-14, *c*-17); *DHA*, docosahexaenoic acid (C22:6 *c*-4, *c*-7, *c*-10, *c*-13, *c*-16, *c*-19); *DPA*, docosapentaenoic acid (C22:5 *c*-7, *c*-10, *c*-13, *c*-16, *c*-19); *SFA*, saturated FA; *MUFA*, monounsaturated FA; *PUFA*, polyunsaturated FA; *HUFA*, highly unsaturated FA (double bonds ≥ 4); n-3, omega-3 fatty acids; n-6, omega-6 fatty acids; HUFAn-3, highly unsaturated FA n-3; *CLA*, conjugated linoleic acids; *SCFA*, short chain FA (≤ C10); *OCFA*, odd chain FA.

^a-b^LSMeans in a row without a common superscript differ (*p* < 0.05).

### Factorial discriminant analysis

**Figure 1:**
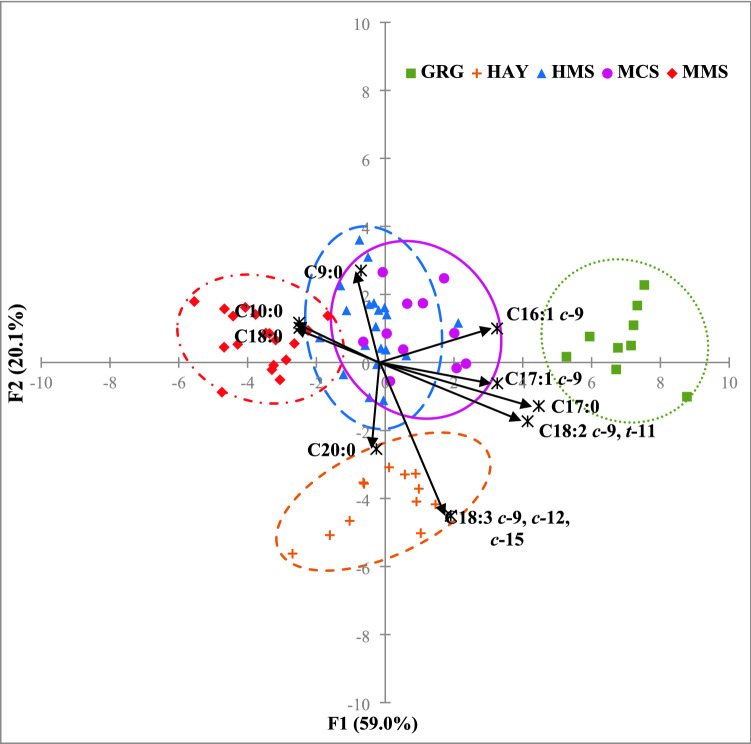
FDA scatterplot of the milk samples according to the five feeding groups based on the fatty acid profiles. The main function F1 (along x-axis) and F2 (along y-axis) accounted for 59.0% and 20.1% of the total variance, respectively. The 0.95 confidence ellipses are drawn around each centroid of groupings. High maize silage (HMS): blue dotted line and blue upward triangles; medium maize silage (MMS): red dotted-pointed line and red diamond symbols; mixed crop silages (MCS): solid purple line and purple closed circles; grass and lucerne hays (HAY): orange dotted line and orange plus symbols; green grass (GRG): green pointed line and green closed squares. The black arrows indicate the most significant (*p* < 0.05) discriminative FA that had correlation coefficient values higher than 0.20 with at least either F1 or F2 (for graphic purposes these significant correlations coefficients were multiplied 10 times according to the maximum value of F1 and F2).

The factorial discriminant analysis (FDA) resulted in two main significant functions (F1 and F2; Wilks’s *λ* = 0.002), accounting for 59.0% and 20.1% of the total variance, respectively. The FDA identified the 9 most significantly (*p* < 0.05) discriminative FA: C9:0, C10:0, C16:1 *c*-9, C17:0, C17:1 *c*-9, stearic acid, (SA, C18:0), ALA, CLA 9 and C20:0; all of which have a correlation coefficient in absolute value greater than 0.25 with F1 and/or F2. Figure 1 shows a FDA scattergram with these discriminative FA against F1 (x-axis) and F2 (y-axis). The FA contributing most to differentiate FG under FDA were poorly aligned with the those identified to differ by the univariate analysis; indeed, only ALA and CLA9 proved to be significant under both analyses. As reported in Fig. 1, the GRG and HAY milk FA profiles clearly differ from silage-based diet profiles and between each other, although there are considerable overlaps among HMS, MMS and MCS samples. HMS and MCS seemed similar and only partially overlap with the MMS group.

The cross-validation used to assess FDA reliability confirmed the accuracy of this supervised targeted model for the correct classification of milk from HAY and GRG groups (Matthews correlation coefficient values of 1.00), however, there was a noticeable misclassification among the silage-derived milk samples, especially for MCS, with 5 out of 11 samples wrongly assigned to HMS (Table 4). However, if all silage samples were considered as single cluster, as suggested by the FDA, the predictive performances is enhanced.

**Table 4:** Descriptive statistics of the cross-validation based on the leave-one-out criteria of the factorial discriminant analysis (FDA).

Predicted	Actual				
	HMS	MMS	MCS	HAY	GRG
HMS	**19**	1	5	0	0
MMS	0	**17**	0	0	0
MCS	1	0	**6**	0	0
HAY	0	0	0	**12**	0
GRG	0	0	0	0	**9**
**Total**	20	18	11	12	9
Sensitivity	0.95	0.94	0.55	1.00	1.00
Specificity	0.88	1.00	0.98	1.00	1.00
Accuracy	0.90	0.99	0.91	1.00	1.00
Precision	0.76	1.00	0.86	1.00	1.00
MCC	0.79	0.96	0.64	1.00	1.00

HMS, high maize silage; *HMS*, high maize silage; *MMS*, medium maize silage; *MCS*, mixed crop silages; *HAY*, grass and lucerne hays; *GRG*, green grass; MCC = Matthews correlation coefficient.

Bold values represent the samples classified correctly.

### Prediction of milk FA composition

The results from the multiple linear regressions using the most predictive FA for the four forage sources (maize silage, other silages, hays, fresh grass) are reported in Table 5. Indeed, although both silages (maize and ‘others’) slightly influence individual FA concentrations, they significantly increased total SFA and, consequently, reduced PUFA, especially ALA and CLA9. Hay feeding is positively correlated with C17:0 and CLA9 and negatively with SA and concentrations, resulting in higher PUFA. Hay also seems to both decrease LA and increase SFA, even though the effect on SFA is weaker than with silages, especially those from mixed-crops. Feeding fresh grass seems to modify the FA profile mildly, even if it too contributes to higher concentrations of two beneficial FA—C17:0 and CLA9. In the case of OCFA there were no significant predictive capacity by any of the roughage sources.

**Table 5:** Multiple linear regression equations of the most discriminant milk fatty acids (FA) and their derived chemical classes based on the dietary forage sources (% DM).

Fatty acids	Intercept	Regression coefficients of the forages				*p* value
		Maize silage	Other silages	Hays	Fresh grass	
C16:1	1.53 (± 0.23)	ns	ns	ns	ns	0.229
C17:0	0.35 (± 0.06)	0.0018^†^	ns	0.0024*	0.0026*	0.001
C18:0	11.4 (± 1.0)	ns	ns	− 0.011^†^	− 0.009^†^	0.078
LA	2.47 (± 0.33)	ns	− 0.018*	− 0.017*	ns	0.001
ALA	0.51 (± 0.11)	− 0.0043*	− 0.0033*	ns	ns	0.005
CLA9	0.53 (± 0.12)	− 0.0026^†^	− 0.0025^†^	0.0027*	0.0041*	0.003
SFA	65.5 (± 1.4)	0.068*	0.084*	0.054*	ns	0.055
MUFA	28.7 (± 1.3)	ns	ns	− 0.045*	ns	0.034
PUFA	5.77 (± 0.47)	− 0.018*	− 0.035*	0.019*	ns	0.001
CLA	0.72 (± 0.13)	− 0.0035*	− 0.0049*	ns	ns	0.001
OCFA	2.13 (± 0.40)	ns	ns	ns	ns	0.699

Each equation (data of FA are as g/100 g of total detected fatty acids) is presented in the following format: intercept of the model (± standard error) and regression coefficient of the forages, when significant (*p < 0.05; ^†^*p* < 0.10; ns = *p* > 0.10). The *p* value refers to the significance of the regression model. LA = linoleic acid, C18:2 *c*-9, *c*-12; *ALA* = alpha linolenic acid, C18:3 *c*-9, *c*-12, *c*-15; CLA9 = C18:2 *c*-9, *t*-11. For the other fatty acids abbreviations see Table 3.

## Discussion

The study was designed to model milk FA responses, in high genetic merit dairy cows under lowland field conditions. Univariate statistical analysis assessed the main differences in milk production, quality and FA profile between the feeding groups, then a supervised factorial discriminant modelling identified the FA *fingerprinting* of the forage groups and finally, the multiple linear regression equations verified the magnitude of the relationships between discriminant FA and forage types.

As expected, the herds’ productive performance were closely linked to feed consumption or DMI, although the potential production of herds feeding maize-silage might have been strategically limited to enhance milk quality and qualify the farms’ destination to protected designation of origin (PDO) for hard cheese production. The lower milk CP, particularly casein content, in GRG samples may be due to an inbalance in ruminal degradability for highly fermentable fibre and N-sources, typically seen for leafy grass consumption. Moreover, the low CP and casein in GRG-milk could also be due polyphenol oxidase activity (from red clover and other legumes) reducing protein degradability in the rumen, and hence amino acid supply, although this is speculative^37^. The lower lactose content in milk from GRG cows, could be due to a combination of lower concentrate supplementation and total feed intake, potentially leading to lower rumen propionate synthesis compared with other groups^22^. Since propionate is the main precursor of ruminant gluconeogenesis, this can lead to a less glucose and hence lactose synthesis. Another explanation might relate to milk yield^38^, as higher milk intermarry pressure (for the other forage groups), can increase milk lactose concentration. In contrast to other studies^12,39^, total milk fat was similar across all forage types, probably a reflection lack of variation in the concentration of antilipogenic FA, such as the rumen intermediate trans C18:1 isomers like C18:1 *t*-10.

Major differences between the FG existed for the concentration of most nutritionally relevant FA, driven by the amount and type of forage in the diets. These often reached significance between GRG and HMS milk, although in some cases GRG milk also differed from MMS, MCS and/or HAY groups with no consistency in the pattern of variation seen for the individual FA. One noticeable outcome from our study is a significant (*p* < 0.05) effect of hay and fresh-grass feeding on the concentrations of total CLA, CLA9 and its precursor VA in milk (especially if compared to HMS), in line with previous studies^9,13,16^. Indeed, dairy diets based on fresh grass have led to significantly higher concentrations in VA, CLA9 and total CLA^40^. Many studies report that forage from permanent meadows produces milk with more CLA and n-3 than from maize or other cereal silage diets^41–43^, as in our survey. These elevated concentrations of CLA are likely to be due to polyphitic forage, rich in LA and ALA, which undergo incomplete hydrogenation, generating the intermediate VA rather SA (C18:0). Both hydrogenation products (VA and SA) are subsequently desaturated in the mammary gland producing CLA9 and oleic acid (OA, C18:1 *c*-9) respectively into milk^44^. As in this study, Akbaridoust et al*.*^12^, confirmed lower milk CLA9 concentrations from partially replacing lowland grazing with maize silage. Both HAY and GRG forages in this study originated as polyphitic vegetation from permanent meadow also leading to higher concentrations of ALA, EPA and n-3 compared with other diets, although differences between GRG and other groups do not always reach significance. Compared to grass only silage diets, the inclusion of red clover silage in TMR-fed cows has led to significantly more n-3 in milk, especially as ALA^45^.

Feeding maize silage as the dominant roughage in this study caused higher concentrations of SCFA (C4:0 and C6:0) and PA, increasing total SFA in milk compared with other FG. Maize silage also elevated n-6:n-3 ratios (mostly driven by lower n-3 rather than more n-6) which is common with other studies^10,19^, however not all findings here are in line with previous reports. With respect to C4:0 and C6:0, Yang et al.^46^, report increasing maize silage reduced their concentrations although another Coppa et al.^47^, highlighted increased de novo-synthesized FA (from C4:0 to C14:0) with reduced consumption of fresh grass (replaced with maize silage) in the cows’ diet. Moreover, this latter study observed a significant increase in the proportion of C8:0 to C12:0 with higher inclusion of maize silage and concentrates in the TMR. Other studies also report greater secretion of PA in milk^13,14,18^ with increasing inclusion of maize silage, although Liu et al. report this major SFA was not influenced by the relative proportions of maize and grass silage in a broad study under natural uncontrolled conditions^21^. Short chain and some medium chain FA are mainly produced by de novo synthesis in the mammary gland, using acetate and butyrate from the ruminal fibrolytic bacteria activity, although some (particularly PA) can be derived directly from the diet, especially with greater use of maize silage^19,48^. With the exception of the HAY group, the NDF content (an indication of digestible fibre) of all diets tended to be remarkably consistent, with mean levels ranging from 36.8 to 37.7%. This might explain why SCFA were not lower with maize silage although does not explain the apparent slightly higher de novo synthesis compared with other forages. Milk SCFA content has been demonstrated to related to grass botanical origin rather than dietary NDF content per se, confirming that mammary de novo FA synthesis could be affected by the proportion of unsaturared FA, probably ruminal biohydrogenation intermediates^39,49^.

This study aimed to evaluate the influence of the feeding system on the lipidic fingerprinting in milk, considering TMR diets with five main roughage sources. Thus, a factorial discriminant analysis (FDA) was carried on the 70 milk FA profiles to identify changes when maize silage was replaced with a mix of other ensiled, dried or fresh forages. Figure 1 confirms HAY-milk samples correlate with ALA (C18:3 *c*-9, *c*-12, *c*-15 on the chart), proving once more to be a specific strong biomarker of hay-based diets^20,21^, with a minor contribution of the long chain SFA C20:0. On the other hand, GRG milk seemed to be characterized by more CLA9 (C18:2 *c*-9, *t*-11 on the chart) and C17:0, even if these FA also discriminate HAY samples. Both FA have previously been identified as biomarkers of fresh grass-based milk by Butler et al*.*^40^ and Paredes et al*.*^16^, respectively. As discussed, the discriminative capacity of CLA9 is likely to be due to the incomplete rumen hydrogenation of dietary LA and ALA and subsequent desaturation in the mammary gland. The odd chain FA C17:0 is derived largely from the rumen microbial activity and its transfer into milk is reported to be enhanced for cows fed hays and fresh grass rich in C18 FA^16,17^—similar to grasses and legume species fed to the HAY and GRG cows in our study. C16:1 *c*-9 was identified as a weak lipidic biomarker of MCS and HMS milk samples, with only minor discriminative capacity, slightly correlated with F1 and, as with C17:1 *c*-9, it appears associated with both MCS and GRG milk. However, explaining their discriminative roles is not easy. From the literature, C16:1 *c*-9 seems to indicate both the use of maize-based diets^18^ and the adoption high concentrates diets^20^, whereas C17:1 *c*-9 has been reported to be associated with fresh grass feeding^18^ and both are the result of Δ9-desaturation (of C16:0 and C17:0) in the mammary gland. Milk from the three FG feeding silages tended to have similar FDA loadings making them spatially overlapping as a single cluster in the left-centre of the scattergram, associated with C9:0, C10:0 and SA. However, MMS group is slightly separated from the other two because of the influence of C10:0 and C18:0 (SA). Other studies report, compared to rations based on a fresh grass, feeding highly digestible silages, seemed to increase the proportion of SFA, such as C10:0^19,20^ and SA^12^, due to a more extensive ruminal biohydrogenation. Although univariate analysis did not detect any significant difference between FG for SA (C18:0) (Table 3), the strong correlation with MMS could be explained by the highest milk production by this group, possibly causing a slightly negative energy balance and release of SA from mobilized body fat^50^.

Extending the findings of the multivariate discriminant model to the large-scale distribution maybe effective to identify dairy products based on, at least, the three feeding strategies investigated in the present study: ensiled (HMS, MMS, MCS) vs. dried (HAY) vs. fresh (GRG) forages; even if they are all produced in the same geographic area. Thus proving the effective role of FA profile to trace the dairy products according to feeding system. Indeed, milk FA profile can be a powerful, reliable and accurate metabolomics tool to discriminate production rations high in cereal-derived silages or a mix of grass and legume-derived hays, which affect the nutritional value of the resulting milk (incidence of beneficial FA), contamination risk (i.e., presence of clostridium bacteria) and the sustainability of system (ratio between input and output of human edible energy).

**Figure 2:**
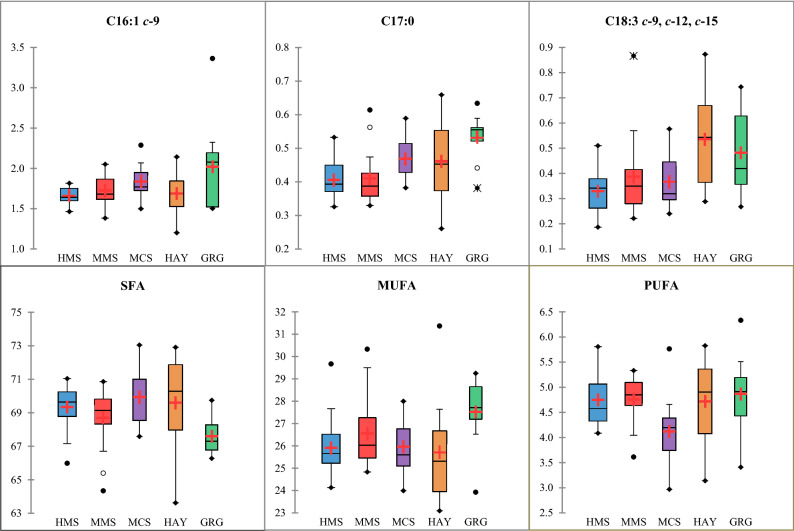
Box–Whisker plots of fatty acids (g/100 g of total fatty acids) according to the five feeding groups. *HMS*, high maize silage; *MMS*, medium maize silage; *MCS*, mixed crop silages; *HAY*, grass and lucerne hays; *GRG*, green grass. The box plots represent the following descriptive statistics: median (bar in box), mean (**+**, red cross), 25% (Q1) and 75% (Q3) quartile (bottom and top end of the box), minimum [Q1 − 1.5 × (Q3 − Q1)] and maximum [Q1 + 1.5 × (Q3 − Q1)] whiskers (lines outside), minimum and maximum values (•, full black circles), outliers with a distance to box of 1.5–3.0 times interquartile range (°, empty circles) or higher than 3 times interquartile range (*, asterisks). For the fatty acids abbreviations see Table 3.

The findings discussed already are mostly confirmed by the stepwise regression models. Indeed, although both maize and others silages slightly influence individual FA, they significantly increased total SFA and, consequently, reduced PUFA, especially for ALA and CLA9 concentrations. An overview of the predictive regression results seemed to highlight the main consequence of replacing maize or other silages with hay or fresh grass is the increase in PUFA beneficial for human health especially CLA and ALA. However, Fig. 2 also shows how variable the concentrations of ALA and total PUFA in milk from the HAY group are, probably because of the range in botanical composition and forage maturity at harvest across the farms throughout the study. Furthermore, feeding silages, especially from cereals other than maize, seem to increase the SFA content of milk, more than hay does, shown by their higher regression coefficients. In contrast to the variability within the HAY and GRG groups, Fig. 2 shows silage-based diets to be more uniform. The feeding system with the greatest effect on milk FA composition is GRG; increasing beneficial FA, such as CLA and C17:0 probably due to the contrasting impact of fresh grass and fermented silages on the rumen activities. As discussed, variability in forages within GRG records might explain outliers within this group (Fig. 2).

Since milk lipid composition depends on a combination of feeding, genetics, stage of lactation and seasonal variation^51^, accurate prediction of FA profile from these forage types is challenging and hard to interpret due to variability in botanical origin, maturation stage and conservation method, not to mention feeding management^41^. However, this study underlines the support that can come from multivariate approaches to predict milk lipidic nutrients, which can be bigger than that coming from univariate models based on farming variables. Further work in this area ought to allow a comprehensive whole system modelling of milk FA origin, accounting for dietary intake, rumen fermentation and hydrogenation, body fat mobilization, de novo mammary lipogenesis and desaturation.

## Conclusions

This study confirms the scope to assess differences in milk FA profiles according to forage type for highly productive dairy cows. Factorial discriminant analysis (FDA) chemometric approach highlights substantial differences in milk composition from cows fed silage diets compared to hays (HAY) or fresh grass (GRG), both of which lead to higher concentrations of FA beneficial for health (e.g. C17:0, ALA and CLA9). Cross-validation confirmed the accuracy of FDA modelling to discriminate HAY and GRG milk samples, although did show mild misclassification for milk from different silage-based diets. Compared with maize silage, milk from perennial swards poliphytic forage seems to be characterised by a greater variability in FA profiles. To summarise, replacing maize silage with hays and/or fresh grass in TMR dairy diets improves the nutritional quality of milk by reducing SFA increasing CLA and long chain PUFA n-3—potentially improving the nutritional sustainability of the dairy products from intensive lowland systems. The study also identified which FA could benchmark biomarkers to distinguish the feeding systems, especially differentiating the use of maize silage.

## Supplementary Information

Supplementary Information.
