# Supplementary material for: Multivariate modelling of milk fatty acid profile to discriminate the forages in dairy cows’ ration
Source: Sci Rep. 2021 Dec 1;11:23201. doi: 10.1038/s41598-021-02600-9 (PMC8636629; doi:10.1038/s41598-021-02600-9)
Supplement: Supplementary file 1 — Supplementary Information. [file 41598_2021_2600_MOESM1_ESM.docx]

| Fatty acids | Dietary forage group | | | | | SEM | p-value |
| --- | --- | --- | --- | --- | --- | --- | --- |
|  | HMS  (*n* = 20) | MMS  (*n* = 18) | MCS  (*n* = 11) | HAY  (*n* = 12) | GRG  (*n* = 9) |  |  |
| C4:0 | 3.20^a^ | 2.97^ab^ | 3.01^ab^ | 3.10^ab^ | 2.73^b^ | 0.108 | **0.024** |
| C5:0 | 0.042 | 0.031 | 0.049 | 0.050 | 0.036 | 0.010 | 0.502 |
| C6:0 | 2.30^a^ | 2.20^ab^ | 2.21^ab^ | 2.18^ab^ | 2.11^b^ | 0.046 | **0.045** |
| C7:0 | 0.036 | 0.037 | 0.053 | 0.031 | 0.053 | 0.009 | 0.267 |
| C8:0 | 1.33 | 1.30 | 1.30 | 1.26 | 1.21 | 0.036 | 0.213 |
| C9:0 | 0.043 | 0.041 | 0.040 | 0.029 | 0.037 | 0.006 | 0.396 |
| C10:0 | 2.99 | 3.03 | 2.92 | 2.76 | 2.70 | 0.102 | 0.122 |
| C11:0 | 0.276 | 0.277 | 0.269 | 0.277 | 0.278 | 0.018 | 0.997 |
| C12:0 | 3.54 | 3.55 | 3.47 | 3.27 | 3.19 | 0.134 | 0.233 |
| C13:0 | 0.188 | 0.237 | 0.186 | 0.178 | 0.181 | 0.020 | 0.076 |
| C14:0 | 11.6 | 11.8 | 11.7 | 11.6 | 11.2 | 0.260 | 0.468 |
| C14:1 *c*-9 | 0.876 | 0.925 | 0.969 | 0.908 | 0.913 | 0.048 | 0.463 |
| C15:0 | 1.09 | 1.17 | 1.22 | 1.13 | 1.09 | 0.065 | 0.384 |
| C15:1 *c*-9 | 0.026 | 0.041 | 0.042 | 0.038 | 0.043 | 0.008 | 0.392 |
| C16:0 | 32.7^ab^ | 32.2^ab^ | 33.3^a^ | 33.2^a^ | 31.2^b^ | 0.663 | **0.026** |
| C16:1 | 1.67 | 1.65 | 1.71 | 1.71 | 1.84 | 0.095 | 0.505 |
| C16:1 *t*-9 | 0.308 | 0.307 | 0.269 | 0.305 | 0.292 | 0.020 | 0.577 |
| C17:0 | 0.409 | 0.422 | 0.473 | 0.460 | 0.502 | 0.028 | 0.117 |
| C17:1 *c*-9 | 0.144 | 0.151 | 0.168 | 0.187 | 0.225 | 0.023 | 0.114 |
| C18:0 | 9.55 | 9.62 | 9.44 | 9.83 | 10.3 | 0.488 | 0.532 |
| C18:1 *c*-9 | 19.5 | 19.3 | 19.0 | 19.5 | 20.8 | 0.504 | 0.090 |
| C18:1 *c*-11 | 0.375 | 0.364 | 0.334 | 0.366 | 0.386 | 0.030 | 0.797 |
| C18:1 *c*-12 | 0.268^a^ | 0.298^a^ | 0.243^ab^ | 0.167^b^ | 0.219^ab^ | 0.024 | **0.002** |
| C18:1 *c*-13 | 0.053 | 0.067 | 0.068 | 0.054 | 0.058 | 0.010 | 0.778 |
| C18:1 *c*-15 | 0.052 | 0.083 | 0.060 | 0.066 | 0.073 | 0.009 | 0.061 |
| ΣC18:1 (*c*-14 + *t*-16) | 0.231 | 0.265 | 0.222 | 0.200 | 0.215 | 0.026 | 0.383 |
| ΣC18:1 *trans* (*t*-6 + *t*-7 + *t*-8) | 0.230 | 0.225 | 0.237 | 0.225 | 0.175 | 0.027 | 0.423 |
| C18:1 *t*-9 | 0.168 | 0.196 | 0.187 | 0.154 | 0.176 | 0.019 | 0.466 |
| C18:1 *t*-10 | 0.302 | 0.317 | 0.262 | 0.274 | 0.356 | 0.051 | 0.239 |
| C18:1 *t*-11 | 0.702^b^ | 0.787^ab^ | 0.782^ab^ | 0.954^ab^ | 1.074^a^ | 0.085 | **0.021** |
| ΣC18:1 *trans* (*t*-12 + *t*-13 + *t*-14) | 0.344^a^ | 0.315^ab^ | 0.266^ab^ | 0.241^b^ | 0.246^ab^ | 0.029 | **0.037** |
| C18:1 *t*-15 | 0.201 | 0.198 | 0.189 | 0.197 | 0.176 | 0.019 | 0.881 |
| C18:2 *c*-9, *c*-12 | 1.97^ab^ | 1.95^ab^ | 1.88^ab^ | 1.62^b^ | 2.13^a^ | 0.162 | 0.066 |
| C18:2 *c*-9, *c*-15 | 0.038 | 0.044 | 0.028 | 0.049 | 0.037 | 0.007 | 0.281 |
| C18:2 *c*-12, *c*-15 | 0.041 | 0.048 | 0.034 | 0.049 | 0.035 | 0.007 | 0.309 |
| C18:2 *c*-9, *t*-11 | 0.366^b^ | 0.409^b^ | 0.378^b^ | 0.442^ab^ | 0.619^a^ | 0.042 | **0.001** |
| C18:2 *c*-9, *t*-12 | 0.081 | 0.091 | 0.061 | 0.082 | 0.095 | 0.010 | 0.123 |
| C18:2 *c*-9, *t*-13 | 0.111 | 0.126 | 0.092 | 0.074 | 0.091 | 0.014 | 0.060 |
| C18:2 *c*-12, *t*-9 | 0.054 | 0.078 | 0.058 | 0.048 | 0.047 | 0.010 | 0.095 |
| C18:2 *c*-13, *t*-8 | 0.041 | 0.048 | 0.033 | 0.034 | 0.040 | 0.005 | 0.166 |
| C18:2 *c*-13, *t*-11 | 0.026 | 0.019 | 0.025 | 0.031 | 0.022 | 0.004 | 0.383 |
| C18:2 *c*-15, *t*-11 | 0.067^b^ | 0.094^ab^ | 0.080^ab^ | 0.106^a^ | 0.110^a^ | 0.012 | **0.041** |
| C18:2 *t*-9, *t*-12 | 0.136 | 0.131 | 0.084 | 0.116 | 0.122 | 0.020 | 0.334 |
| C18:2 *t*-10, *t*-14 | 0.119 | 0.132 | 0.130 | 0.140 | 0.163 | 0.015 | 0.306 |
| C18:2 *t*-11, *t*-15 | 0.050 | 0.071 | 0.056 | 0.046 | 0.058 | 0.010 | 0.330 |
| C18:3 *c*-9, *c*-12, *c*-15 | 0.343^b^ | 0.430^ab^ | 0.365^ab^ | 0.513^a^ | 0.487^ab^ | 0.048 | **0.045** |
| C18:3 *c*-6, *c*-9, *c*-12 | 0.032 | 0.037 | 0.033 | 0.037 | 0.037 | 0.006 | 0.877 |
| C18:3 *c*-9, *c*-11, *c*-15 | 0.059 | 0.059 | 0.042 | 0.164 | 0.164 | 0.009 | 0.164 |
| C18:3 *c*-9, *c*-13, *c*-15 | 0.030 | 0.041 | 0.037 | 0.041 | 0.049 | 0.005 | 0.595 |
| C19:1 | 0.171 | 0.188 | 0.172 | 0.191 | 0.165 | 0.021 | 0.833 |
| C20:0 | 0.112 | 0.135 | 0.128 | 0.142 | 0.131 | 0.013 | 0.395 |
| C20:1 *c*-8 | 0.087 | 0.085 | 0.081 | 0.100 | 0.101 | 0.009 | 0.384 |
| C20:2 *c*-11, *c*-14 | 0.056 | 0.052 | 0.046 | 0.054 | 0.059 | 0.007 | 0.732 |
| C20:3 *c*-8, *c*-11, *c*-14 | 0.116 | 0.123 | 0.107 | 0.096 | 0.103 | 0.010 | 0.265 |
| C20:3 *c*-11, *c*-14, *c*-17 | 0.029 | 0.035 | 0.033 | 0.029 | 0.027 | 0.005 | 0.776 |
| C20:4 *c*-5, *c*-8, *c*-11, *c*-14 | 0.141^ab^ | 0.165^a^ | 0.160^a^ | 0.116^b^ | 0.140^ab^ | 0.009 | **0.007** |
| EPA | 0.090^b^ | 0.097^ab^ | 0.091^ab^ | 0.124^a^ | 0.127^a^ | 0.010 | **0.021** |
| C22:0 | 0.073 | 0.075 | 0.067 | 0.097 | 0.091 | 0.010 | 0.148 |
| C22:1 *c*-13 | 0.038 | 0.050 | 0.035 | 0.037 | 0.038 | 0.005 | 0.150 |
| C22:2 *c*-13, *c*-16 | 0.0451 | 0.052 | 0.039 | 0.054 | 0.042 | 0.008 | 0.573 |
| C22:3 *c*-13, *c*-16, *c*-19 | 0.038 | 0.047 | 0.044 | 0.035 | 0.034 | 0.005 | 0.231 |
| C22:4 *c*-7, *c*-10, *c*-13, *c*-16 | 0.043 | 0.048 | 0.051 | 0.041 | 0.043 | 0.006 | 0.772 |
| DHA | 0.046 | 0.043 | 0.041 | 0.047 | 0.035 | 0.005 | 0.456 |
| DPA | 0.092 | 0.113 | 0.077 | 0.079 | 0.091 | 0.011 | 0.187 |
| C23:0 | 0.034^b^ | 0.061^a^ | 0.051^ab^ | 0.050^ab^ | 0.051^ab^ | 0.007 | **0.051** |
| C24:0 | 0.033 | 0.031 | 0.026 | 0.031 | 0.033 | 0.005 | 0.875 |
| C24:1 *c*-15 | 0.041 | 0.039 | 0.042 | 0.036 | 0.036 | 0.005 | 0.872 |

Table S1. Effect of dietary roughage source (forage group) on milk fatty acid profile (g/100 g of total quantified fatty acids). HMS = high maize silage; MMS = medium maize silage; MCS = mixed crop silages; HAY = grass and lucerne hays; GRG = green grass; SEM = standard error of the means. Fatty acids abbreviations: EPA = eicosapentaenoic acid (C20:5 *c*-5, *c*-8, *c*-11, *c*-14, *c*-17); DHA = docosahexaenoic acid (C22:6 *c*-4, *c*-7, *c*-10, *c*-13, *c*-16, *c*-19); DPA = docosapentaenoic acid (C22:5 *c*-7, *c*-10, *c*-13, *c*-16, *c*-19).
